# Sun-related knowledge and practices in Irish construction and agricultural workers

**DOI:** 10.1093/occmed/kqae042

**Published:** 2024-07-11

**Authors:** V Hogan, M Hogan, O Kirwan, C Langan Walsh, C McLaughlin, Á Moynihan, A Connolly, J Walsh, M Coggins

**Affiliations:** Discipline of Health Promotion, School of Health Sciences, Áras Moyola, University of Galway, H91TK33, Galway, Ireland; School of Psychology, University of Galway, H91TK33 Galway, Ireland; School of Natural Sciences & Ryan Institute, University of Galway, H91TK33 Galway, Ireland; Discipline of Health Promotion, School of Health Sciences, Áras Moyola, University of Galway, H91TK33, Galway, Ireland; School of Natural Sciences & Ryan Institute, University of Galway, H91TK33 Galway, Ireland; School of Natural Sciences & Ryan Institute, University of Galway, H91TK33 Galway, Ireland; UCD Centre for Safety & Health at Work, School of Public Health, Physiotherapy, and Sports Science, University College Dublin, D04V1W8 Dublin, Ireland; School of Natural Sciences & Ryan Institute, University of Galway, H91TK33 Galway, Ireland; School of Natural Sciences & Ryan Institute, University of Galway, H91TK33 Galway, Ireland

## Abstract

**Background:**

Agricultural and construction workers spend much of their work time outdoors and have higher risks of developing skin cancer when compared to indoor workers. However, there is limited research on ultraviolet radiation (UVR) exposure knowledge, sun safety practices and constraints within these occupational groups in Ireland.

**Aims:**

This study aimed to examine self-reported time spent outdoors in a sample of Irish agricultural and construction workers; to describe and compare UVR exposure knowledge, safety practices and perceived constraints in both occupational groups, and to assess the association of demographic, personal and occupational factors with sun-related knowledge, practices and perceived constraints.

**Methods:**

Agricultural workers (*n* = 154) and construction workers (*n* = 467) completed a questionnaire, which measured solar UVR exposure knowledge, safety practices, and perceived constraints to sun personal protective equipment and sunscreen use in addition to demographic, personal, and workplace characteristics. Mann–Whitney and Kruskal–Wallis tests were used to examine differences in knowledge, practices and perceived constraints by these characteristics.

**Results:**

Both groups spend a significant proportion of their working week outdoors (25 hours per week on average). Although participation in sun safety training was high for both groups, UVR exposure knowledge and sunscreen use were low, and annual rates of reported sunburn were high. Knowledge, practices and perceived constraints also differed significantly according to demographic, personal, occupational and workplace characteristics.

**Conclusions:**

In addition to training by employers and advisory groups, interventions are required to address perceived barriers that impede the uptake and usage of control measures that can lower risk.

## Teaser

This study reports results from a survey of sun safety knowledge and practice among Irish construction and agricultural workers. Across both sectors, workers spend an average of 25 hours outdoors. Most workers received sun safety training, but sunscreen application and use of sun-protective clothing are low. Awareness of measures such as the ultraviolet index or sun protection factor is low. Interventions to promote safe sun behaviours must address constraints to the use of protective measures.

**Key learning points**

**What is already known about this subject:**

- Sun exposure and sun-protective behaviours have not been examined in agricultural or construction workers in Ireland.

**What this study adds:**

- Although levels of sun-protective training were high, reported protective behaviours could be improved.
- While construction workers report more constraints preventing personal protective equipment use and sunscreen use, agricultural workers report lower ultraviolet-radiation-related knowledge.

**What impact this may have on practice or policy:**

- Advisory bodies and employers should concentrate efforts on the design of interventions to reduce perceived barriers to protective actions and continue to provide regular sun-protective training to outdoor workers.

## Introduction

Skin cancer is the third most prevalent form of invasive cancer in Ireland; currently, more than 13 000 cases are diagnosed annually, with rates expected to double by 2040 [1]. Solar ultraviolet radiation (UVR) exposure and sunburn are among the top risk factors in the development of skin cancer in Ireland [2]. While most workers may experience solar UVR exposure on an intermittent or short-term basis during their working day, outdoor workers are more exposed than indoor workers [3–5]. Research examining occupational solar UVR exposure in Ireland is limited, with only two previous studies, examining exposure among golf course workers [6] and gardeners [7]. Data on solar UVR exposure among construction and agricultural workers are available for many EU countries [8–11], but there have been no such studies conducted in Ireland to date. Solar UVR levels in Ireland are typically in the moderate range during the summer months and range on average between 240 and 1668 J/m^2^, from June to December [12]. Solar UVR levels in Ireland tend to be much lower than observed in, for example, Mediterranean countries such as Spain [13]. Although the temperate Irish climate may lead some employers and employees to downplay the risk associated with solar UVR exposure, evidence suggests that this view is mistaken. Complete cloud cover reduces UVR by 50%; therefore, outdoor workers may still be exposed [5,14]. Importantly, climate change and its impact on outdoor workers, in terms of more frequent heatwaves and higher levels of solar UVR, have been key drivers for increased focus on the working conditions of outdoor workers [5,8]. In Ireland, recent statistics highlight that 23% of skin-cancer-related deaths occur among farmers, construction and outdoor workers [15]; therefore, it is timely to estimate exposure levels and investigate worker knowledge, practices and perceived constraints to sun-protective behaviours within these sectors so that targeted interventions can be designed to reduce risk.

## Methods

A convenience sample of construction and agricultural workers was employed in this study. Before data collection, ethical approval for this study was achieved using a research ethics checklist developed for MSc dissertations and approved by the academic staff supervising the research. Permission to distribute questionnaires was sought and granted in advance of the questionnaire distribution. Study information sheets and paper-based questionnaires were distributed to 633 construction workers who were working on construction sites or attending apprenticeship schools in Ireland, resulting in a response rate of 75% (*n* = 477). Ten questionnaires were discarded as unusable, reducing the available sample to *n* = 467. Study information sheets and electronic questionnaires were distributed to 250 farmers attending a distance education course, achieving a response rate of 62% (*n* = 154). An overall usable sample of 621 study participants was achieved. Table 1 displays the sample demographic and worker characteristics in both occupational groups. Most participants were male (86%); however, there were more female participants in the agriculture group (38%) than in the construction group (4%). In both groups, the largest proportion of participants were aged between 21 and 30 years. The majority in both groups were White, and 32% of construction workers and 54% of farmers reported having type I/II skin types, using the Fitzpatrick scale [16].

**Table 1.** Sample demographics and time worked outdoors

		Construction workers, *n* (%)	Farmers, *n* (%)
Gender (*n* = 620)	Male	444 (95)	95 (62)
	Female	18 (4)	59 (38)
	Other	4 (1)	
Age (*n* = 615)	<20	48 (10)	9 (6)
	21–30	191 (41)	57 (37)
	31–40	85 (19)	27 (18)
	41–50	82 (18)	11 (7)
	51–60	43 (9)	27 (18)
	61+	14 (3)	21 (14)
Ethnicity (*n* = 618)	White	431 (93)	154 (100)
	BAME	33 (7)	0
Skin complexion (*n* = 615)	White, very pale	36 (8)	52 (34)
	White, pale	111 (24)	30 (20)
	White–light brown	204 (44)	56 (36)
	Medium brown	85 (18)	16 (10)
	Dark brown	15 (3)	0
	Black	10 (2)	0
Work type (*n* = 620)	Mainly indoors	206 (44)	129 (84)
	Equally indoors/outdoors	93 (20)	24 (15)
	Mainly outdoors	167 (36)	1 (1)

		Mean (SD)	Mean (SD)
Hours worked outdoors (*n* = 614)	All participants	25 (20)	26 (15)
	Worked mainly indoors	11(14)	23 (10)
	Worked mainly outdoors	41 (17)	25
	Equally indoors and outdoors	24(14)	41 (25)

Total sample *N* varies slightly across variables due to missing data.

Four measures adapted from previous studies were included in the questionnaire: sun safety attitudes (four items) [17], solar UVR knowledge (four items) [18], perceived sunscreen use constraints (five items) [18], and perceived sun-protective clothing constraints (six items) [18]. The sun safety attitudes measure, solar UVR knowledge measure, perceived use of sunscreen constraints and perceived sun-protective clothing constraints measure all employed five-point Likert scales ranging from ‘strongly disagree’ to ‘strongly agree’. Higher scores indicated better attitudes, better knowledge and higher perceived constraints. Sun-protective behaviours [18] were examined using eight single-item questions, examining protective clothing use (one item), removal of protective clothing (two items), sunscreen use (four items) and checking UV index (one item). A personal protective equipment (PPE)-protective behaviour measure was generated by calculating a composite score based on the use of three pieces of sun-protective PPE: a long-sleeved top, hat and neck scarf. A sun-protective behaviour measure was generated by calculating a composite score for two items: the use of sunscreen and checking the UV index. Copies of the questionnaires employed are available in Supplementary File 1 (available as Supplementary data at *Occupational Medicine* Online).

All descriptive and inferential statistics were conducted using SPSS, Version 26. Means and standard deviations were calculated for continuous variables, and frequencies/percentages were calculated for categorical level data. Chi-square analyses were conducted to examine nominal level differences between the two occupational groups. Mann–Whitney and Kruskal–Wallis tests were conducted to compare differences in the dependent variables of attitudes, knowledge, perceived constraints and protective behaviours by personal, occupational and work-related features. Post hoc pairwise comparisons for significant Kruskal–Wallis test statistics were generated as part of the SPSS output.

## Results

Table 1 presents the proportion of workers who worked indoors, outdoors or a combination of both. Most farmers (84%) reported working mainly indoors, while 44% of construction workers also reported working mainly indoors. Reported hours spent outdoors ranged from 11 to 41 hours, with an average of 25 and 26 hours for construction and agricultural workers, respectively. Over one-third of construction workers indicated that they mainly worked outdoors, on average 41 hours per week. Table 2 presents sun-protective behaviours and training. Just over half of all survey participants reported using sunscreen at work; however, rates of sunscreen use during overcast or rainy weather were much lower than during sunny weather. Half of construction workers and 55% of farmers reported that they only applied sunscreen once per day. Almost two-thirds of participants (63%) reported sunburn experiences as part of an average working year, with farmers significantly more likely than construction workers to report being sunburnt, χ^2^ (1, *N* = 614) = 76.643, *P* < 0.001. Checking of the UV index was very low in both occupational groups, and significantly lower in the farmer group, χ^2^ (2, *N* = 618) = 67.431, *P* < 0.001. Participation in training and/or receiving information relating to exposure to solar UVR was high amongst both groups. However, when compared with construction workers, a significantly higher proportion of farmers reported that previous training/information had aided their understanding of the risks associated with sun exposure, χ^2^ (2, *N* = 497) = 36.787, *P* < 0.001.

**Table 2.** Sun-protective behaviours and training

		Construction workers, *n* (%)	Farmers, *n* (%)
Sunburn (*n* = 614)**	Yes	246 (53)	141 (93)
	No	216 (47)	11 (7)
Sunscreen use at work (*n* = 620)	Yes	241 (52)	78 (51)
	No	225 (48)	76 (49)
When sunny (*n* = 621)	Yes	244 (52)	103 (67)
	No	223 (48)	51 (33)
When overcast (*n* = 621)	Yes	29 (6)	45 (29)
	No	438 (93)	109 (71)
When raining (*n* = 618)	Yes	10 (2)	35 (23)
	No	454 (98)	119 (77)
Sunscreen re-application rate (*n* = 619)	Hourly	6 (2)	1 (1)
	Every 2–3 hours	78 (29)	26 (28)
	Less than every 3 hours	52 (19)	15 (16)
	Once per day	271 (50)	51 (55)
Check UV index (*n* = 620)***	Agree	83 (18)	0 (0)
	Neutral	108 (23)	9 (6)
	Disagree	273 (59)	145 (94)
Sun safety training/ information received (*n* = 621)	Yes	305 (65)	112 (75)
	No	162 (35)	42 (25)
Training aids understanding of risk***	Yes	222 (58)	92 (82)
(*n* = 497)	No	65 (17)	20 (18)
	Don’t remember	98 (25)	0 (0)
Remove long sleeve top during sunny weather (*n* = 621)***	Never	113 (24)	25 (16)
	Seldom	53 (11)	40 (26)
	Sometimes	133 (29)	40 (26)
	Often	87 (19)	40 (26)
	Always	81 (17)	9 (6)

Chi-square test: *P < 0.05, **P < 0.01, ***P < 0.001.

**Figure 1.**
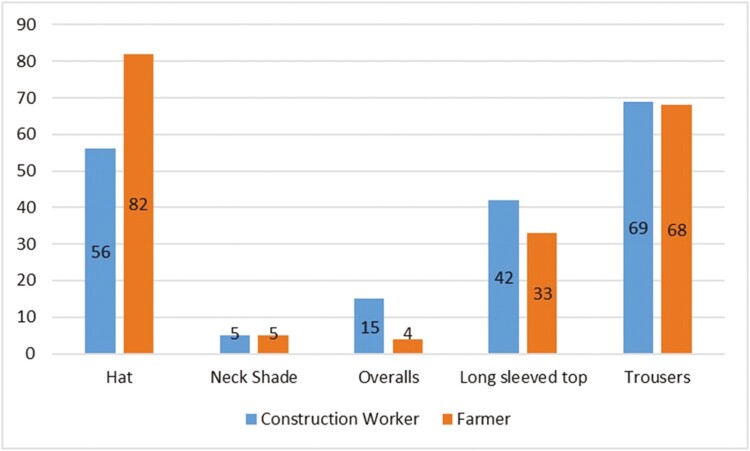
Reported sun-protective clothing worn during sunny weather.

Figure 1 displays the sun-protective clothing choices made by both occupational groups during sunny weather. Percentage frequencies are presented for each clothing item, based on a ‘tick all that apply’ question format. Protective hats and wearing of trousers were the most common protective clothing choices reported, while wearing of neck shades was less frequent. The use of neck shades was equally low among both groups. Construction workers and farmers differed significantly in the practice of removing their shirt/top during sunny weather, χ^2^ (4, *N* = 621) = 33.730, *P* < 0.001, with a higher proportion of construction workers reporting that they always remove their top/shirt in sunny weather (see Table 2).

Table 3 shows significant Chi-square test differences between the two occupational groups on each of the four items measuring attitudes towards sun protection. Farmers were less likely to understand the meaning of sun protection factor (SPF) and were less likely to value wearing sunglasses to protect their eyes. Farmers were also less likely to think that sun protection measures were important if the exposure duration was less than 1 hour and were more likely to indicate that sunscreen was not required on overcast/cloudy days.

**Table 3.** Attitudes towards sun protection

		Construction workers, *n* (%)	Agricultural workers, *n* (%)
Understand sun protection factor (SPF) (*n* = 620)**	Agree	377 (81)	46 (30)
	Neutral	38 (8)	11 (7)
	Disagree	51 (11)	97 (63)
Sunscreen is not required on a cloudy/overcast day (*n* = 621)**	Agree	187(40)	126 (82)
	Neutral	111 (24)	19 (12)
	Disagree	169 (36)	9 (6)
It is important to wear sunglasses to protect your eyes from the sun (*n* = 621)**	Agree	359 (77)	36 (23)
	Neutral	70 (15)	33 (21)
	Disagree	38 (8)	85 (56)
Sun protection is important when working outside for less than one hour in the sun (*n* = 618)**	Agree	315 (68)	26 (17)
	Neutral	94 (20)	28 (18)
	Disagree	56 (12)	99 (65)

Chi-square test: *P < 0.05, **P < 0.01.

The results of the Mann–Whitney and Kruskal–Wallis analyses are presented in Table 4, which focused on differences in UVR knowledge, attitudes, sun-protective practices, and perceived PPE and sunscreen constraints (dependent variables), by occupation, hours spent outdoors, age and skin type (independent variables). Construction workers and agricultural workers differed significantly on all six dependent variables. In the two-group Mann–Whitney comparison, construction workers reported significantly better UVR knowledge, attitude, sun PPE behaviour and sun-protective behaviour than agricultural workers, but also significantly higher constraints preventing the use of both sun PPE and sunscreen. In the sample as a whole, participants who engaged in low levels of outdoor work (<10 hours) reported better knowledge and attitudes than those working longer hours outdoors (*P* < 0.05 for all three comparisons); however, they also reported significantly higher sunscreen use constraints (*P* < 0.05 for all three comparisons) and sun-protective PPE constraints than participants who worked outside 11–20 and 21–30 hours, respectively (*P* < 0.05). Participants who worked >31 hours per week outdoors reported significantly better PPE-protective behaviour and better sun-protective behaviour when compared with groups working either 11–20 or 21–30 hours outdoors. Younger workers (<30 years) reported more constraints against the use of sun-protective PPE than workers aged >51 years (*P* < 0.05). Younger workers (<30 years) also reported high sunscreen constraint scores in comparison with the two older age categories (*P* < 0.05). Workers with very pale skin type reported less constraints preventing the use of either sun-protective PPE or sunscreen when compared with those with pale, medium or dark skin type (*P* < 0.05 for all comparisons); however, they also reported lower knowledge when compared with those with pale or dark skin (*P* < 0.05). See Table 4 for all significant post hoc pairwise comparisons.

**Table 4.** Occupational, work-related and personal factors in relation to the six dependent variables

Independent variables	*N*	UVR knowledge score *M* (SD)	*U/K* (Diff†)	UVR Attitude score *M* (SD)	*U/K* (Diff†)	Sunscreen constraint score *M* (SD)	*U/K* (Diff†)	Sun PPE constraint score *M* (SD)	*U/K* (Diff†)	Sun PPE behaviour score *M* (SD)	*U/K*	Sun-protective behaviour score *M* (SD)	*U/K* (Diff†)
Occupation													
Construction		12.70 (3.17)	9.04***	14.51 (2.44)	12.83***	13.17 (3.77)	13.26***	16.21 (4.21)	10.52***	1.82 (1.11)	2.17*	0.70 (0.66)	2.81**
Agriculture		9.64 (4.50)		8.87 (4.15)		8.23 (2.94)		12.06 (3.32)		1.63 (1.11)		0.51 (0.50)	
Hours outdoors													
1. 0-10	182	12.93 (3.28)	35.95***	14.44 (3.15)	44.38***	12.99 (4.15)	35.15***	15.84 (4.50)	23.92***	1.85 (1.01)	15.62**	0.51 (0.64)	26.96***
2. 11–20	120	11.32 (4.01)	(1–2†)	12.17 (4.41)	(1–2†)	10.96 (4.41)	(1–2, 2–4†)	14.38 (4.42)	(1–2, 2–4†)	1.53 (1.12)	(1–2†)	0.63 (0.55)	(1-2, 1-4†)
3. 21–30	95	10.53 (4.45)	(1–3, 2–3†)	11.05 (4.41)	(1–3†)	10.54 (3.95)	(1–3, 3–4†)	13.75 (4.15)	(1–3, 3–4†)	1.56 (1.11)	(1–3†)	0.57 (0.60)	
4. >31	192	12.02 (3.50)	(1–4, 3–4†)	13.43 (3.08)	(1–4, 3–4†)	12.17 (3.85)	(1–4†)	15.69 (4.15)		1.96 (1.17)	(2–4, 3–4†)	0.83 (0.63)	(2-4, 3-4†)
Age													
1. Under 30 years	289	11.81 (3.71)	NS	13.35 (3.58)	NS	12.37 (4.34)	11.18**	15.88 (4.77)	17.95***	1.71 (1.10)	NS	0.64 (0.61)	NS
2. 31–50	197	12.29 (3.72)		13.30 (3.90)		11.76 (4.05)		14.74 (3.92)	(1–2†)	1.82 (1.12)		0.64)	
3. >51	99	11.54 (4.12)		12.11 (4.23)		10.89 (3.72)	(1–3†)	13.96 (3.70)	(1–3†)	1.89 (1.12)		0.63 (0.62)	
Skin type													
1. Very pale	85	11.21 (4.94)	8.35*	12.29 (4.54)	NS	9.68 (4.45)	33.24***	12.65 (4.61)	30.09***	1.67 (1.05)	NS	0.69 (0.56)	NS
2. Pale	132	12.15 (3.64)	(1-2†)	13.23 (3.81)		12.46 (4.04)	(1–2†)	15.56 (4.45)	(1–2†)	1.85 (1.05)		0.64 (0.64)	
3. Medium	248	11.77 (3.48)		13.09 (3.80)		11.98 (3.96)	(1–3†)	15.24 (4.18)	(1–3†)	1.76 (1.15)		0.66 (0.63)	
4. Dark	120	12.43 (3.68)	(1-4†)	13.64 (3.22)		12.63 (3.94)	(1–4†)	16.16 (3.99)	(1–4†)	1.83 (1.16)		0.60 (0.64)	

Skin types listed in Table 4 are derived from the Fitzpatrick scale and amalgamated into four skin types; skin types 3 and 4 on the Fitzpatrick scale were grouped into the ‘Medium’ skin type and skin types 5 and 6 were grouped into the ‘dark’ skin type.

*M* = mean, SD = standard deviation, NS = not significant, *U/K* = Standardized Mann–Whitney *U* (reported for two-group comparisons) or Kruskal–Wallis *K* statistic (reported for >2 groups comparisons).

*P < 0.05, **P < 0.01, ***P < 0.001.

Diff† = Significant group difference (*P* < 0.05) based on pairwise mean rank comparison, post hoc for significant Kruskal–Wallis *K* (reported for >2 groups comparisons).

## Discussion

Construction and agricultural workers in the current study spent a significant proportion of their working time outdoors, 25 hours on average, with some spending up to 41 hours outdoors. Although reported rates of sun safety training were high, sunburn rates were also high and almost half of workers in both groups did not use sunscreen. Solar UVR knowledge, sun exposure attitudes, sun exposure practices and perceived barriers impacting the use of PPE and sunscreen differed significantly between the two occupational groups. Significant differences were observed on a number of these dependent variables related to hours worked outdoors, skin type and age of participants.

This is the first cross-occupational comparison of time worked outdoors among outdoor worker groups in Ireland, examining sun exposure, knowledge, and protective behaviours and perceived constraints in two important sectors of the Irish economy, which employs an estimated 17% of the Irish workforce [19]. Although the study adopted a convenience sampling approach and is not nationally representative, a strength of the study is the high survey response rates of both occupational groups (>60%). Previous surveys of outdoor workers have reported low response rates (e.g.<40%) [6,20]. This study also provides more detailed information on sunscreen usage among outdoor workers than previous Irish studies, with useful information on the use of sunscreen in different weather conditions and re-application patterns examined.

There are several limitations in our study. As the study used self-reported measures only, there is potential for common method variance influencing the results and some results may have been subject to recall bias. A convenience sampling approach was employed, which limits the generalizability of the results. Future studies examining solar UVR exposure levels would benefit from concurrent collection of personal UVR exposure data using standard erythemal dose (SED) monitors [21], in addition to self-report data. Several important factors were not examined in this study, that is, seasonal variation in exposure, the timing of exposure during working hours and other precautionary measures such as checking for moles, wearing sunglasses, and use of sources of shade. Future studies in Ireland should include these aspects of exposure and sun-protective behaviour.

The self-reported working time outdoors, of approximately 25 hours per week, supports previous research, which has established that outdoor workers including construction and agricultural workers are potentially exposed to higher doses of UVR compared to indoor workers [4,5,10]. The average outdoor working hours reported in this study are comparable to those reported in a nationwide German study of outdoor workers across various professions, who reported an average of 24 hours outdoors per week [8]. The rate of reported sunburn was high at 63% and compares unfavourably to Schneider *et al*. [8] who reported that one-fifth of workers experienced at least one sunburn in the past year. Although agricultural workers were significantly more likely to report sunburn than construction workers, the results overall reflect insufficient sun-protective behaviour within both occupational groups. In Ireland, most of the population has a fair complexion [22,23] and is therefore at higher risk of developing non-melanoma skin cancer (NMSC) [24]. Notably, just under one-third of construction workers and over half of agricultural workers in this study indicated that they had a fair complexion. However, it has been noted in one previous study that outdoor workers may incorrectly rate their skin complexion [25]. Previous research indicates that construction workers and agricultural workers do not use sunscreen [11]. In this study, roughly half of the participants in both occupations indicated that they used sunscreen, which may suggest a trend towards improved awareness over time. Nevertheless, the sunscreen application rate is lower than that reported by the general public (68% in Ireland) [26]. In addition, the self-reported sun-protective clothing choices in this study, including the limited use of full protective clothing and the practice of removing tops when working in sunny conditions indicate focal points for behaviour change interventions.

Construction workers in this study reported significantly better solar UVR knowledge, attitudes, sun-protective PPE use and behaviour than agricultural workers. However, construction workers also reported a significantly higher level of perceived constraints to the use of both sun-protective PPE (e.g. time consuming, interferes with work) and sunscreen use at work (e.g. difficult to apply, uncomfortable, time consuming). To improve sun protection behaviour, these findings suggest that perceived constraints are an important focal point for intervention in the construction sector. Previous research demonstrates that perceived constraints or barriers have a negative influence on behaviour change [27] and that barriers to sun protection have the strongest impact on sun-protective behaviour [28,29]. Consultation with construction workers in relation to the types of sun-protective PPE and sunscreen available on site may aid in the identification of more suitable options and enhance usage.

Although the training levels reported in this study compare favourably with previous Irish research focused on golf course workers [6], the findings indicate room for improvement. For example, while agricultural workers reported to a significantly higher extent than construction workers that training aids their understanding of sun risks, their attitudes to protective behaviours were significantly lower than construction workers. This finding may reflect limitations in training initiatives within this sector. Employers in both sectors should make use of freely available resources from regulatory and healthcare bodies. For example, the Health Service Executive’s Sunsmart initiative [30] provides evidence-based videos, posters and guidance documents for outdoor workers. These resources along with supplementary training, monitoring and feedback are recommended to promote positive behaviour change. Furthermore, research suggests that farmers prefer practical as opposed to lecture-led training [31]; therefore, the incorporation of sun safety training into farm walks and farm visits by farming bodies is recommended.

Participants with pale skin reported significantly fewer barriers to the use of both sun-protective clothing and sunscreen. This suggests that workers who are more vulnerable to the effects of solar UVR may be more aware of their susceptibility and may be more amenable to the use of protective measures. Given the susceptibility of all outdoor workers, risk-reduction interventions should address how barriers to sun-protective behaviour are perceived by different skin type groups and seek to reduce barriers for all workers. Younger workers in this study also reported significantly more barriers to using sun-protective PPE and sunscreen than older workers. Therefore, induction training of young apprentices in the construction sector and young agricultural workers should concentrate on raising awareness, whilst re-fresher training, toolbox talks, farm safety events and adequate supervision of young workers are required to reinforce practice.

John *et al*. [32] have called for global action to address the rate of NMSC in outdoor workers. This is currently important in Ireland where there is limited information on exposure and protective behaviour within the construction and agricultural sectors. The cross-comparative design adopted in the current study not only allows an initial estimation of exposure in both occupational groups, but also highlights risk factors such as low knowledge and perceived constraints that operate across both groups. These baseline findings highlight areas for improvement for both sectors, and the need for enhanced national initiatives to promote sun-protective behaviour, which can be acted upon by employers and by advisory bodies such as the Construction Industry Federation and Teagasc (Irish semi-state body responsible for training and advisory services in the agri-food sector). Based on the findings from this study, there are different training needs in each of the sectors. Training initiatives in the agricultural sector should focus initially on raising awareness of the basics of sun safety protection, while both sectors should focus on enhancing attitudes and addressing constraints to sun-protective behaviour.

Going forward, the use of SED measurement is required to establish occupational exposure to solar UVR in Ireland. These direct measures can be used in conjunction with self-reported data on sun-protective behaviours to estimate risk and this form of quantitative analysis coupled with the design of effective risk-reduction interventions is an important avenue of work to combat rising rates of NMSC. It has been noted that the lack of regulation for solar UVR is in part due to a lack of sufficient data on which to distinguish occupational and non-occupational exposure [24].

In conclusion, continued focus is needed on occupational exposure to solar UVR to accurately establish the risk of skin cancer in outdoor workers. In the absence of legislation governing solar UVR exposure, evidence-based recommendations on protecting outdoor workers are vital.

## Supplementary Material

kqae042_suppl_Supplementary_File_1
