# Supplementary material for: Sun-related knowledge and practices in Irish construction and agricultural workers
Source: Occup Med (Lond). 2024 Jul 11;74(5):378–85. doi: 10.1093/occmed/kqae042 (PMC11285147; doi:10.1093/occmed/kqae042)
Supplement: kqae042_suppl_Supplementary_File_1 [file kqae042_suppl_supplementary_file_1.doc]

Investigating Solar UV Radiation: Exposure Times, Behaviours and

Attitudes Among Irish Construction and Farming Workers[[1]](#footnote-2)

Questionnaire Survey

This study aims to evaluate the ultraviolet radiation (UV) an outdoor construction worker may receive from the sun during a normal working day as well as the sun safety knowledge and behaviour which influences this exposure**.**

**I have read and understood the participant information sheet and consent to participate in this study** Yes ☐ No ☐

**About You** (Please tick or complete the response where indicated)

1. Your Gender - Male ☐ Female ☐ Other ☐

1. Age - less than 20 years ☐ 21-30 years ☐ 31-40 years ☐ 41-50 years☐ 51-60 years ☐ 61 years or older ☐

1. Ethnicity - White Irish /European ☐ Asian ☐ Black / African / Caribbean ☐ Mixed / Multiple ethnic groups ☐ Other ethnic group_________________________

1. Occupation (Construction)

Roofer ☐ Labourer ☐ Machinery Operator ☐ Painter ☐ Brick Layer ☐

Engineer ☐ Plasterer ☐ Landscaper ☐ Supervisor/Foreman ☐ Glazier ☐

Scaffolder ☐ Other Please Specify _________________

Occupation (Farming) - Please indicate the type of farming you are engaged in (Tick all that apply)

Dairy ☐ Beef ☐ Sheep ☐ Poultry ☐ Tillage ☐ Mixed ☐

Other Please Specify _________________

1. How long have you been working in this industry? ______________ years

**Section 1: Sun Exposure**

1. During your regular working week, how many hours do you spend outdoors?

____________ hours

1. How many times in a typical working year have you suffered from sun burn as a result of workplace exposure?

__________ times

1. Do you work mostly indoors or outdoors? (Please tick one)

Only indoors ☐ Mostly indoors ☐ Equal amounts of indoor and outdoor ☐ Mostly outdoors ☐ Only outdoors ☐

1. Please indicate your level of agreement with the following statements

|  | Strongly Disagree | Disagree | Neither  Agree nor Disagree | Agree | Strongly Agree |
| --- | --- | --- | --- | --- | --- |
| I like to have a suntan | □ | □ | □ | □ | □ |
| I usually check the UV index forecast | □ | □ | □ | □ | □ |

**Section 2 Complexion**

1. Please tick which skin complexion you are most like

| 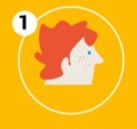 | 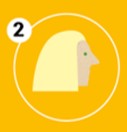 | 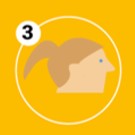 | 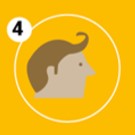 | 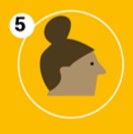 | 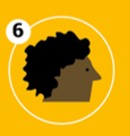 |
| --- | --- | --- | --- | --- | --- |
| **Skin Type 1**  Skin Colour-  Very pale white, Pale white with  freckles. | **Skin Type 2** Skin ColourPale White. | **Skin Type 3**  Skin Colour- White, light brown. | **Skin Type 4**  Skin Colour- Medium brown, dark brown. | **Skin Type 5**  Skin Colour- Dark Brown | **Skin Type 6**  Skin Colour-  Black |
| ☐ | ☐ | ☐ | ☐ | ☐ | ☐ |

1. Please tick your tanning ability

| Burns very easy, never tans. | Burns easily, rarely tans | Sometimes burns, gradual tan | Hardly ever burns, tans easily | Rarely burns, tans easily and darkens quickly | Never burns, tans very easily and very dark very quickly |
| --- | --- | --- | --- | --- | --- |
| ☐ | ☐ | ☐ | ☐ | ☐ | ☐ |

**Section 3 – Sun Exposure Protective Behaviour**

1. Which of the following items do you wear at work during sunny weather? (Tick all that apply)

Hat ☐ Neck shade ☐ Overalls ☐ Short sleeved top ☐

Long sleeved top ☐ Trousers ☐ Shorts ☐ None of the above ☐

1. During warm weather do you remove your long-sleeved shirt to accommodate your work (Please tick one)

| Never | Seldom | Sometimes | Often | Always |
| --- | --- | --- | --- | --- |
| ☐ | ☐ | ☐ | ☐ | ☐ |

1. During warm weather do you remove any other protective items, as listed in question 1, to accommodate your work (Please tick one)

| Never | Seldom | Sometimes | Often | Always |
| --- | --- | --- | --- | --- |
| ☐ | ☐ | ☐ | ☐ | ☐ |

1. Do you use sunscreen at work? Yes ☐ No ☐

If yes what Sun Protection Factor (SPF)? (i.e. SPF 15, 20, 30, 50, 50+)

_____________

1. Do you use sunscreen at work when the weather is….. (Please tick all that apply)

Sunny ☐ Overcast ☐ Raining ☐ I don’t wear sunscreen ☐

1. When do you put on sunscreen (Please complete a-f and tick one option per section)

|  |  | Yes | No |
| --- | --- | --- | --- |
| (a) | At home before I leave for work | ☐ | ☐ |
| (b) | In the car before I start work | ☐ | ☐ |
| (c) | As soon as I feel the sun/heat of the sun on my skin | ☐ | ☐ |
| (d) | After being in the sun for a while, but before I realise that my skin has turned red | ☐ | ☐ |
| (e) | If I realise that my skin has turned red | ☐ | ☐ |
| (f) | I don’t’ wear sunscreen | ☐ | ☐ |
|  |  |  |  |

1. How often would you say, you re-apply sunscreen? (Please tick one)

| a) About once an hour | ☐ |
| --- | --- |
| b) Every 2-3 hours | ☐ |
| c) Less than every 3 hours | ☐ |
| d) I only apply once a day | ☐ |
| e) I don’t wear sunscreen | ☐ |

1. Do you use sunbeds?
   1. Yes ☐
   2. No ☐

1. If you answered yes to **Q8**, please specify how often you use sunbeds

1. Weekly ☐
2. Monthly ☐
3. Yearly ☐

**Section 4: Attitudes to Sun Exposure**

1. Please indicate your level of agreement with the following statements (Please complete a-d and tick one option per section)

|  |  | Strongly disagree | Disagree | Neither Agree nor Disagree | Agree | Strongly Agree |
| --- | --- | --- | --- | --- | --- | --- |
| (a) | I understand the meaning of sun protection factor (SPF) - | ☐ | ☐ | ☐ | ☐ | ☐ |
| (b) | I don’t need to wear sunscreen  on a cloudy/overcast  day during summer | ☐ | ☐ | ☐ | ☐ | ☐ |
| (c) | It is important to wear sunglasses to protect your eyes from the sun. | ☐ | ☐ | ☐ | ☐ | ☐ |
| (d) | Sun protection is important when working outside for less than one hour in the sun. | ☐ | ☐ | ☐ | ☐ | ☐ |

1. For me, using sunscreen at work is … (Please complete a-d and tick one option per section)

|  |  | Strongly Disagree | Disagree | Neither  Agree nor  Disagree | Agree | Strongly Agree |
| --- | --- | --- | --- | --- | --- | --- |
| (a) | Difficult to apply | ☐ | ☐ | ☐ | ☐ | ☐ |
| (b) | Uncomfortable | ☐ | ☐ | ☐ | ☐ | ☐ |
| (c) | Expensive | ☐ | ☐ | ☐ | ☐ | ☐ |
| (d) | Time consuming | ☐ | ☐ | ☐ | ☐ | ☐ |
| (e) | Something that keeps me from getting the tan I want | ☐ | ☐ | ☐ | ☐ | ☐ |

1. For me, wearing sun protection clothes (e.g., long sleeves and long trousers) at work is … (Please complete a-f and tick one option per section)

|  |  | Strongly Disagree | Disagree | Neither  Agree nor  Disagree | Agree | Strongly Agree |
| --- | --- | --- | --- | --- | --- | --- |
| (a) | Difficult | ☐ | ☐ | ☐ | ☐ | ☐ |
| (b) | Uncomfortable | ☐ | ☐ | ☐ | ☐ | ☐ |
| (c) | Embarrassing and awkward | ☐ | ☐ | ☐ | ☐ | ☐ |
| (d) | Interfering to my work | ☐ | ☐ | ☐ | ☐ | ☐ |
| (e) | Part of my daily routine | ☐ | ☐ | ☐ | ☐ | ☐ |
| (f) | Something that keeps me from getting the tan I want | ☐ | ☐ | ☐ | ☐ | ☐ |

**Section 5 – UV Exposure Outcomes**

1. You are exposed to UV radiation when …. (Please complete a-d and tick one option per section)

|  |  | Strongly Disagree | Disagree | Neither Agree nor Disagree | Agree | Strongly Agree |
| --- | --- | --- | --- | --- | --- | --- |
| (a) | When you  are in the shade | ☐ | ☐ | ☐ | ☐ | ☐ |
| (b) | When you  are in the sun at work | ☐ | ☐ | ☐ | ☐ | ☐ |
| (c) | When its cloudy | ☐ | ☐ | ☐ | ☐ | ☐ |
| (d) | When it rains | ☐ | ☐ | ☐ | ☐ | ☐ |

1. (Construction): Please tick which training measures you have received at your place of work in relation to sun exposure and protective measures (Please tick all that apply).

|  | Construction Industry Federation Ireland (CIF) | Health and Safety Authority (HSA) | Employer Organised | Other |
| --- | --- | --- | --- | --- |
| Leaflet | ☐ | ☐ | ☐ | ☐ |
| Signage | ☐ | ☐ | ☐ | ☐ |
| Workshop | ☐ | ☐ | ☐ | ☐ |
| Tool box talk | ☐ | ☐ | ☐ | ☐ |
| Online Course | ☐ | ☐ | ☐ | ☐ |
| Safe Pass Training | ☐ | ☐ | ☐ | ☐ |
| No training received | ☐ | ☐ | ☐ | ☐ |
| Can’t remember | ☐ | ☐ | ☐ | ☐ |

(Farming): Have you ever received any information/education on sun exposure from the following?

Teagasc ☐ Health and Safety workshop ☐ GP Surgery ☐ Other, please specify _________________

1. If you received any educational information, did it add to your understanding of any potential risks associated with sun exposure?

Yes ☐ No ☐

1. From the educational information received, what are the potential health risks associated with outdoor work in the sun? (If you did not receive any training, please leave blank)

_________________________________________________________________________

_________________________________________________________________________

**Thanking you kindly for completing this questionnaire**

1. Note: A unique questionnaire was distributed to construction and farming workers, separately. The questionnaires were identical except for question 4 in About You, and question 2 in Section 5. Unique wording for farming workers are indicated in purple font. [↑](#footnote-ref-2)
